# Exiting the COVID-19 pandemic: after-shock risks and avoidance of disruption tails in supply chains

**DOI:** 10.1007/s10479-021-04047-7

**Published:** 2021-04-05

**Authors:** Dmitry Ivanov

**Affiliations:** Berlin School of Economics and Law, Department of Business and Economics, Professor of Supply Chain and Operations Management, 10825 Berlin, Germany

**Keywords:** Supply chain resilience, Supply chain dynamics, Supply chain risk management, Simulation, Ripple effect, COVID-19 pandemic, Disruption tail

## Abstract

Entering the COVID-19 pandemic wreaked havoc on supply chains. Reacting to the pandemic and adaptation in the “new normal” have been challenging tasks. Exiting the pandemic can lead to some after-shock effects such as “disruption tails.” While the research community has undertaken considerable efforts to predict the pandemic’s impacts and examine supply chain adaptive behaviors during the pandemic, little is known about supply chain management in the course of pandemic elimination and post-disruption recovery. If capacity and inventory management are unaware of the after-shock risks, this can result in highly destabilized production–inventory dynamics and decreased performance in the post-disruption period causing product deficits in the markets and high inventory costs in the supply chains. In this paper, we use a discrete-event simulation model to investigate some exit strategies for a supply chain in the context of the COVID-19 pandemic. Our model can inform managers about the existence and risk of disruption tails in their supply chains and guide the selection of post-pandemic recovery strategies. Our results show that supply chains with postponed demand and shutdown capacity during the COVID-19 pandemic are particularly prone to disruption tails. We then developed and examined two strategies to avoid these disruption tails. First, we observed a conjunction of recovery and supply chain coordination which mitigates the impact of disruption tails by demand smoothing over time in the post-disruption period. Second, we found a gradual capacity ramp-up prior to expected peaks of postponed demand to be an effective strategy for disruption tail control.

## Introduction

Historically, manufacturing companies have been exposed to different risks, long before the term “supply chain” has been coined. Disruptions encountered can be both instantaneous, triggered by some single-point-failure interruptions in material flows (e.g., fires or tsunamis) and long-term crises such as pandemics, financial or political crises, and wars. One peculiarity of the COVID-19 pandemic as a massive disruption is that this has been the first long-term supply chain crisis for the last decades which were characterized by transformations of production from insourcing to outsourcing, from local to global, and from redundant to lean. In addition, these decades of relative stability in demand and supply led to formation of crisis-free management mentality, belief in having risks and uncertainty under control, long-term planning, rigid and lean network structures and planning paradigms—it was all turned upside down during the COVID-19 pandemic. The pandemic challenged supply chain management by novel and distinct context of order and chaos, controllable and uncontrollable, rigid and fluid, fixed and adaptable, and certain and uncertain.

Entering the COVID-19 pandemic wreaked havoc on supply chains (Ivanov & Dolgui, 2020a, 2020b, 2021; Queiroz et al. 2020). Reacting and adapting to the pandemic conditions have been challenging tasks (El Baz & Ruel, 2021, Paul and Chowdhury et al. 2021, Ivanov 2021b, Wieland, 2021). While the research community has made considerable efforts to predict the pandemic’s impacts (Ivanov, 2020a) and examine supply chain adaptive behaviors during the pandemic (Ivanov, 2021b; Yang et al. 2021), little is known about supply chain management during pandemic elimination and post-disruption recovery.

**Fig. 1:**
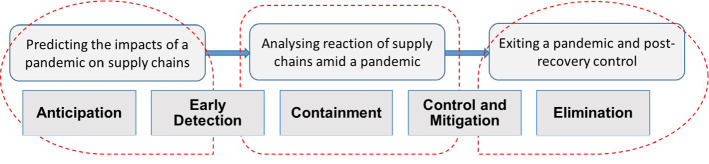
Timing of supply chain disruption management decisions through a pandemic

Ivanov (2020b), Ivanov and Dolgui (2020b), Hosseini et al. (2020), Ruel et al. (2021) and Wieland and Durach (2021) call for re-thinking and extending supply chain resilience from a closed-loop, equilibrium-search based analysis toward viability and transformation perspectives and so motivating our focus on the adaptation stage when transiting from a pandemic (i.e., a “new normal”) toward a post-pandemic recovery (i.e., a “post-new normal”). Figure 1 illustrates the timing of the decisions regarding guiding supply chains (or bouncing them forward) after a long-term disruption crisis.

Some research hypothesizes delayed effects of the COVID-19 pandemic leading to supply chain disorders and the associated need for a balanced approach to exiting the pandemic and recovery management (Paché, 2020). In particular, a pandemic exit could be a challenging task for many supply chains because of after-shock effects such as “disruption tails”. Disruption tails arise in a post-disruption period and are the postponed effects of demand–supply mismatches during a disruption (Ivanov, 2019). Ivanov (2021c) defines a disruption tail as “*a postponed effect of a residue from a disruption period, such as backlog and delayed orders, which appears in the post-disruption / recovery period and may influence supply chain operations and performance even after the disruption recovery. For example, a highly excessive inventory and destabilization of inventory dynamics can be observed after a capacity disruption recovery as a consequence of backlog orders if an inventory control policy is not adapted accordingly*.”

Disruption tails are related to supply chain resilience and recovery management (Dolgui et al. 2020b, Dubey et al. 2019, 2021; Ghadge et al. 2013; Gupta et al. 2020; He et al. 2019; Hosseini et al. 2019, 2020, Li et al. 2020, 2021; Sawik, 2019, 2020; Xu et al. 2020). One major reason for disruption tails is caused by different timing of ordering and production capacity management decisions. While ordering decisions are driven by actual demand and can be adjusted quickly, production capacity management decisions are less responsive and usually use demand forecasts. If capacity and inventory management is unaware of the disruption tails, this can result in highly destabilized production–inventory dynamics and decreased performance in the post-disruption period. Besides, disruption tails can lead to increased costs in the supply chain (Aldrighetti et al. 2021).

Disruption tails have been recently observed in the wake of instantaneous (i.e., singular event-driven) supply chain disruptions and studied in conjunction with the ripple effect, i.e., disruption propagation across different supply chain echelons (Dolgui et al. 2020a; Ivanov, 2020c; Li et al. 2021; Llaguno et al. 2021). However, literature is silent about disruption tails in particular and after-shock risks in the decision-making settings with long-term, massive disruptions in general. Motivated by this research gap, we identify three major research questions (RQ) for our study as follows:

RQ1: What after-shock risks can be encountered in supply chains according to different demand and capacity management dynamics during the pandemic?

RQ2: Why different demand and capacity management dynamics during the pandemic can lead to the after-shock risks?

RQ3: How can the firms avoid negative consequences of the disruption tails and associated after-shock risks when exiting the pandemic?

In answering these RQs, we build on and extend the existing knowledge by constructing and using a discrete-event simulation model to investigate recovery strategies for supply chains in the context of the COVID-19 pandemic. This study makes two contributions to the existing research on exiting disruption periods. First, we examine disruption tails in the pandemic context, unlike other studies, which considered them in the context of instantaneous disruptions. Second, we define and test two recovery strategies: demand smoothing for the pandemic elimination period and a gradual capacity ramp-up for supply chain adaptation to the post-pandemic period.

The paper is organized as follows: In Sect. 2, we review the relevant existing literature. Section 3 is devoted to description of our model, assumptions, and experimental results. In Sect. 4, we elaborate on theoretical, managerial, and policy-making implications derived from our study. Finally, in Sect. 5, we conclude the paper by summarizing its major insights and outlining future research directions.

## Literature review

### Disruption tails

Simulation is a powerful and practice-oriented technique for studying the dynamics of complex supply chains. A considerable body of research can be found in the literature on supply chain simulation and disruption management (Carvalho et al. 2012; Dolgui et al. 2018; Ivanov & Rozhkov, 2020; Lohmer et al. 2020; Machdonald et al. 2018; Schmitt et al. 2017; Schmitt and Sing 2012; Tan et al. 2020; Zhao et al. 2019). Specifically, simulation studies have revealed disruption tails in supply chains (Ivanov 2019; Ivanov and Rozhkov, 2020). For example, a highly excessive inventory and the destabilization of inventory dynamics as a consequence of a backlog of orders can be observed after a capacity disruption recovery if an inventory control policy is not adapted accordingly. Several works (Dolgui et al. 2020a; Ivanov 2019; Ivanov and Rozhkov, 2020) have observed that non-coordinated ordering and production policies during a disruption period may result in backlogs and delayed orders, the accumulation of which causes post-disruption supply chain instability, resulting in further delivery delays and non-recovery of supply chain performance. These residues have been named “disruption tails.” The extant literature suggests that, to avoid these disruption tails, specific “revival” policies must be developed for the transition from recovery to disruption-free operation. However, these insights are limited to instantaneous disruptions (i.e., singular events such as tsunamis or fires) and have not been studied in a pandemic setting so far. Our study makes a distinct and substantial contribution to remedy this gap in the literature.

### The COVID-19 context in supply chain research

Supply chain operations during pandemic times are characterized by a long-term disrupted state in the supply network, an unstable current situation, and uncertainty about future developments in the markets, supply base, and capacities (Sodhi et al. 2021). Together these characteristics entail a danger of supply chain collapses and interruption of the provision of goods and services (Alikhani et al. 2021; Golan et al. 2020; Govindan et al. 2020). The existing research on the pandemic’s impacts on supply chain focuses on two areas: how to predict the pandemic’s impacts on supply chains and how to examine supply chain reactions to the pandemic (El Baz & Ruel, 2021; Golan et al. 2020; Ivanov, 2020a; Paul & Chowdhury, 2021; Queiroz et al. 2020; Singh et al. 2021; Yang et al. 2021). In the first published research on the COVID-19 pandemic’s impact on supply chains, Ivanov (2020a) studied how different pandemic scenarios of varying severity and velocity influence connectivity and the ripple effect in global supply chains. The major observation drawn from these simulation experiments is that the timing of the closing and opening of facilities at different echelons in a supply chain is one of the crucial factors determining the pandemic’s impact on that supply chain’s performance. Lead-time, speed of epidemic propagation, and the upstream and downstream disruption durations in a supply chain have also been identified as important for predicting network performance and resilience under uncertainty. Those results were echoed by Singh et al. (2021) who used anyLogistix software to simulate pandemic impacts on India supply chains. Furthermore, Paul and Chowdhury (2021) have studied recovery plans, and Ivanov and Dolgui (2021) have conducted a survey of optimization and simulation method applications in modeling the ripple effects under pandemic conditions.

Moreover, researchers have pointed to the severity and magnitude of the pandemic disruption and called for supply chain viability—“the ability of a supply chain to maintain itself and survive in a changing environment through a redesign of structures and replanning of performance with long-term impacts” (Ivanov & Dolgui, 2020a, 2020b). The Viable Supply Chain model (Ivanov, 2020b) triangulated supply chain management under pandemic conditions and spanned the perspectives of supply chain ecosystems, multi-structural network designs, and viability capabilities.

In summary, our analysis shows that a pandemic represents a specific type of disruption risk—a *super disruption*. Table 1 depicts the four major aspects that characterize super disruptions and differentiate the pandemic disruption from all other “instantaneous” (i.e., an event with immediate impact) disruptions (Choi 2020; Gupta et al. 2021; Ivanov and Dolgui 2020a, b; Ivanov 2020a, b).

**Table 1:** Instantaneous supply chain disruptions and super disruptions (supply chain crises) (Ivanov, 2021c)

	Instantaneous disruption, e.g., an earthquake or fire	Super disruption (supply chain crisis), e.g., a pandemic
Impact	Instant impact	Long-lasting impact with barely predictable scaling
Scope	Single supply chain echelon (with possible propagations)	Simultaneous disruptions in supply, demand, and logistics
Recovery	Begins when the disruption is over	Is performed in the presence of the disruption and its unpredictable scaling
Timing	A single disruptive event	Simultaneous and/or sequential openings and closures of suppliers, facilities, and markets

Supply chain resilience provides a well-developed body of knowledge of how to manage disruptions that are considered short-term events. However, the COVID-19 pandemic is a novel context that requires going beyond an instantaneous event-driven understanding and can be described as a supply chain *crisis.* A *supply chain crisis* is a long-term disrupted state that is characterized by an unstable current situation and uncertainty about future developments in the markets, supply base, and capacities, which together entail a danger of supply chain collapses and interruption of the market provision of goods and services.

Ivanov (2020a) and Paul and Chowdhury (2021) point to a *very long period of disruption* and its unpredictable scaling during a pandemic. Unlike other disruptions, the pandemic’s disruption profile is characterized by gradual degradation and recovery rather than by instant disruptions of high magnitude and immediate reactions, as is the case, for example, for natural disasters. Since the pandemic is long-lasting and its dynamics can be forecasted (e.g., by SIR models), supply chains may have more time to adapt (Ivanov 2021b; Nagurney 2021). In addition, the gradual and long-lasting pandemic disruption profile may allow for the avoidance of disruption tails and overlays and lead to different insights than instant-event disruptions.

Contrary to the instantaneous disruption, *recovery during the pandemic begins in the presence of the disruption* and so is challenged by deep uncertainty about demand and supply (Choi 2021, Mehrotra et al. 2020). This is different from instantaneous disruptions such as earthquakes, which hit the supply chain once, and the recovery begins when the disruption is over (Ivanov 2021a; Lücker et al. 2020). Third, in the pandemic, there are *simultaneous disruptions in demand, supply, and logistics infrastructure*. This is different from classical disruption risks which usually impose shocks on either supply or demand but not both. Fourth, the pandemic is challenging because of the *timing of disruption propagation.* Different supply chain echelons are hit by disruptions (i.e., due to lockdowns and quarantines creating workforce shortages and demand surges) at different times. This is a novel timing setting with simultaneous and/or sequential openings and closures of suppliers, facilities, and markets (Queiroz et al. 2020).

At the same time, some commonalities between instantaneous and pandemic disruptions can be observed. Both instantaneous and pandemic disruptions are characterized by capacity disruption and recovery needs. The pandemic (or at least the first shutdowns) did also have immediate impacts. The difference is that the impact grew, evolved, and continued with no clear end in sight.

Based on the literature analysis and the articulated specifics of the COVID-19 pandemic and associated supply chain crises, we can identify two major research gaps. First, while the prediction of the pandemic’s impacts and examination of supply chain adaptive behaviors during the pandemic have received attention from the research community, little is known about supply chain management during the pandemic elimination and post-disruption recovery periods. Second, disruption tails have been previously studied in the context of instantaneous disruptions, but to the best of our knowledge, there is no published research on disruption tails in the pandemic context. We take up these research opportunities to develop a substantial contribution to closing the research gaps identified above.

## Modeling and experiments

### Problem context

**Fig. 2:**
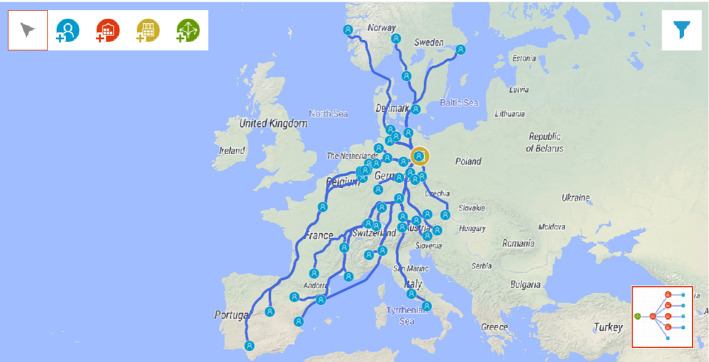
Supply chain design (screenshot from any Logistix Studio software)

Our problem and the simulated supply chain network are based on the real-life context and data of a brewery located in Berlin, which operates a distribution center (DC) in Berlin that serves about 1000 customers across Europe (for simplicity, we reduced the number of customers in the simulation to 50 through some aggregation according to ZIP codes) (Fig. 2).

The whole process from the original brewing to the finished product takes four to six weeks depending on how long each type of beer must be stored. All beer produced is stored in the DC in Berlin. An external service provider is employed for logistics.

**Fig. 3:**
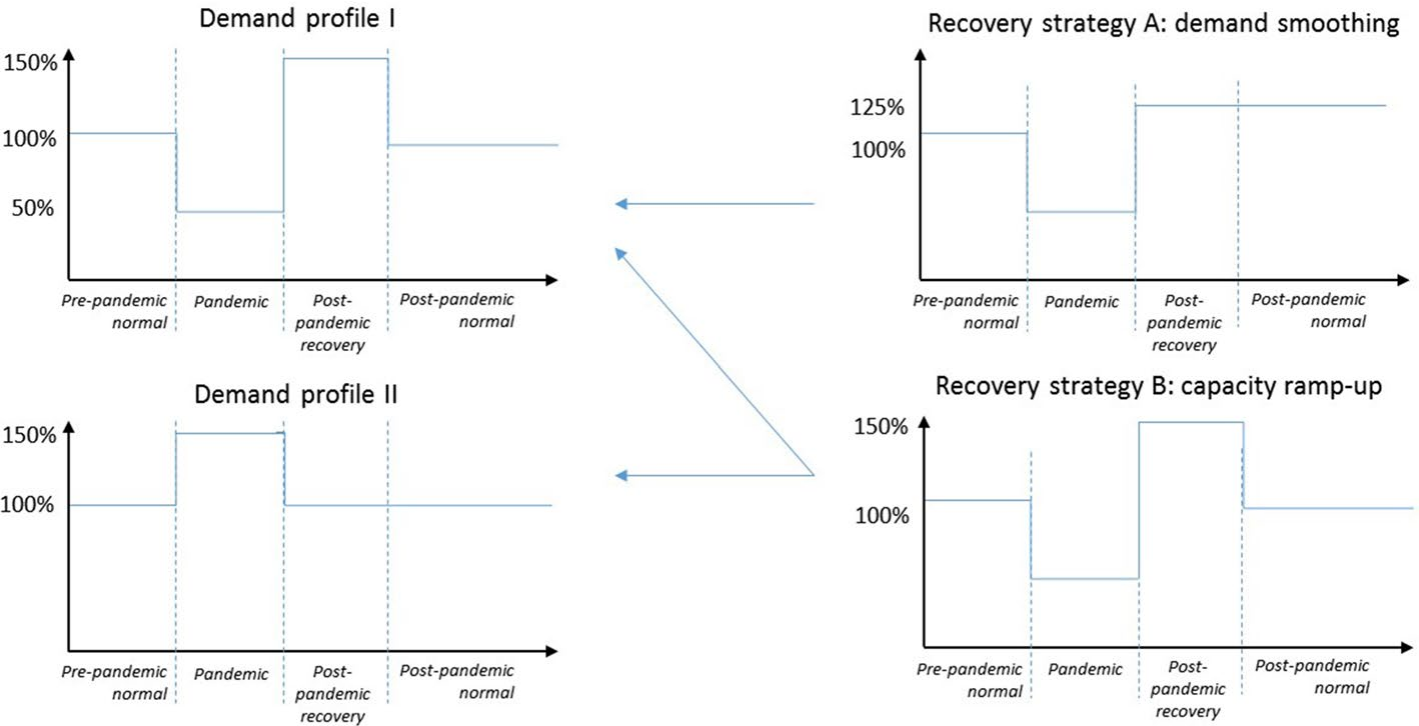
Analysis of pandemic impacts and recovery strategies

The total demand over the simulation period is 5651 beer crates, which is considered 100% (Fig. 3).

Two possible demand scenarios are considered: demand decrease by 50% during the pandemic (profile I) and demand increase by 50% during the pandemic (profile II). In profile I, the demand decline during the pandemic is followed by a demand increase in the post-pandemic recovery period and stabilizes in the post-pandemic normal period at the level of the pre-pandemic normal. In profile II, demand stabilizes at the level of the pre-pandemic normal immediately after the pandemic is over.

Capacity is assumed to be lowered by 50% during the pandemic time period because of government measures to control the epidemic (i.e., lockdowns and quarantines) and the company’s own employee protection measures, which result in 50% availability of the workforce.

Finally, two supply chain recovery strategies are considered: (a) demand smoothing and (b) capacity ramp-up in anticipation of pandemic elimination and post-pandemic recovery. A demand smoothing strategy requires coordination with customers to distribute the orders equally over the post-pandemic recovery and post-pandemic normal periods in order to avoid demand peaks in the post-pandemic recovery period. A capacity ramp-up in anticipation of pandemic elimination and post-pandemic recovery could allow the company to cope with demand peaks. We also consider a combination of these two strategies in the experiments.

### Model and assumptions

**Fig. 4:**
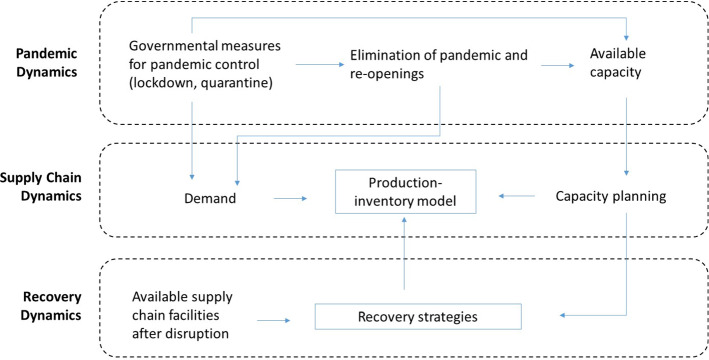
Simulation logic

We developed a discrete-event simulation model using the software anyLogistix. anyLogistix is software that combines simulation and optimization of supply chains, allowing for building a digital supply chain twin for operations and performance analysis under disruptions. Numerous studies (Aldrighetti et al. 2020; Ivanov 2018, 2019, 2020a, c; Singh et al. 2021) utilized anyLogistix in modelling supply chain resilience and disruptions. In Fig. 4, we illustrate the logic of our simulation model.

Because the COVID-19 pandemic is a very special kind of disruption, that is, a long-term supply chain crisis or a super disruption, we address its specific characteristics in our simulation model. First, the pandemic is characterized by a very long-term period of disruption. We account for that in the timeline of our simulation. Second, the pandemic disruption is shaped by disruptions at several supply chain echelons with simultaneous and/or sequential openings and closures of suppliers, facilities, and markets (Ivanov, 2020a). In our model, we consider simultaneous disruptions in demand and capacity. Third, the pandemic disruption begins and ends gradually (unlike instantaneous events) and allows for some time to make decisions about in-advance supply chain capacity adjustments (e.g., pre-positioning extra inventory or ramping-up capacity prior to demand recovery). In our model, we test the recovery strategy when capacity is ramped-up in anticipation of a demand peak after the pandemic as new orders are added to the backlogged demand from the pandemic time.

The simulation model is constructed subject to the following assumptions:

#### Structural dynamics

We assume that the supply chain structure does not change during the pandemic and post-pandemic periods. In other settings, supply chain nodes can completely disappear from the networks (e.g., because of supplier bankruptcy). Since we consider a case with one DC and one production facility, changes in the supply chain structure cannot be applied to our experimental setting.

#### Demand

We do not consider demand variations during each of the periods. This is to avoid unnecessary and inessential randomness, which would bias the output results and make them difficult to interpret correctly. Certainly, this is a contextual limitation of our simulation that might need to be addressed in other industry contexts.

#### Capacity

We allow for capacity decrease and increase by some fixed level. In other analysis settings, the available capacity level can be considered dynamic and dependent on the pandemic dynamics (as captured by SIR modeling) and the forecasted number of infected employees.

#### Pandemic modeling

The pandemic impact is modeled through surges in demand and capacity. Specific pandemic control measures are not considered, but they could influence the intensity of contacts and degree of mobility in society and therefore affect the capacity and demand levels. We also do not account for any pre-pandemic decisions that could be made in anticipation of the pandemic. This assumption is in line with our observations of real companies’ operations—most firms have not developed any resilience measures to cope with pandemics and reacted only once the demand and supply have been disrupted.

#### Inventory and production

The inventory policy at the DC and production facility is a continuous order-up-to-level control policy with a re-order point (also known as *s*, *S* policy). Production control is synchronized with the inventory control policy parameters and driven by them.

### Experiments

#### Parameters

For simulations, data about demand, order frequency, and production-inventory control parameters (i.e., re-order point and target inventory) are needed. Production output is determined by the parameters of production and inventory control. The simulations have been run using the following parameters (Table 2).

**Table 2:** Modeling parameters

Parameter	Parameter value
Total demand in the supply chain per week, in beer crates	5651
Order frequency of customers, in days	7
Re-order point, in crates	500
Target inventory level, in crates	6000
Demand coefficient during the pandemic in profile I	0.5
Demand coefficient during the pandemic in profile II	1.5
Demand coefficient during the post-pandemic recovery in profile I	1.5
Coefficient of inventory control policy parameters during the post-pandemic recovery in the capacity ramp-up recovery strategy	1.5
Demand coefficient during the post-pandemic recovery in the demand smoothing recovery strategy	1.25

We divided the simulations into four periods: pre-pandemic normal, pandemic, recovery after the pandemic, and post-pandemic normal. During the pandemic period, two different demand profiles are considered: an increase by 50% and a decrease by 50%. The capacity is disrupted by 50% during the pandemic period. Studies by Dolgui et al. (2020a), Ivanov (2020c), Ivanov and Rozhkov (2020), Lücker et al. (2020), Sawik (2020), and Singh et al. (2021) show that data about demand and capacity is most crucial to model supply chain disruptions. In combination with the production-inventory control policies, this data can be considered sufficient for disruption modeling and observing meaningful managerial insights. Two recovery strategies—demand smoothing after the pandemic and capacity ramp-up in the wake of pandemic elimination—are tested. The remainder of this section is organized according to these settings.

#### Model validation

**Fig. 5:**
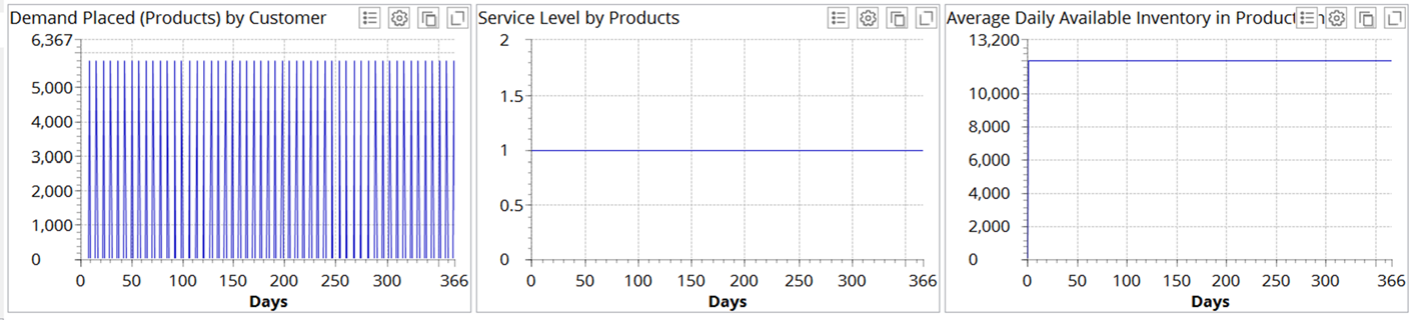
Simulation results for the business-as-a-usual scenario

We validate our model in four different ways. First, we tested the model on the ideal (i.e., business-as-usual) case (Fig. 5).

It can be observed in Fig. 5 that, in the business-as-a-usual scenario, service level (i.e., on-time delivery computed as a ratio of orders delivered on time to total orders placed) in the supply chain is 100%, and the production–inventory system exhibits stable and balanced performance (as seen in the stable daily inventory average). These outcomes confirm the company’s operational results.

Second, we performed a network optimization experiment using data from our simulations. The resulting profit corresponds to the profit in the simulation experiments and the real company’s profit. Third, we visually checked the dynamics of material flows through the simulation. Fourth, a set of variation experiments with different parameters (e.g., re-order point and demand) were performed. They confirmed the model’s sensitivity. Because these sensitivity analysis results do not have any novel managerial implications, we omit their detailed presentation in the paper and focus on major experiments that yield new management insights. Finally, the output data analysis in the log files, replication tests, and warm-up time have been applied for the validity proof. The disruptions have been scheduled in the middle of the simulation period in order to avoid the “noise” at the start of the simulation experiment.

#### Profile I

**Fig. 6:**
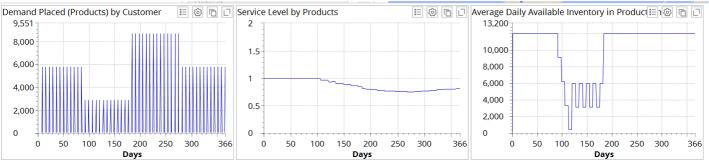
Experimental results for demand profile I when no recovery strategy is deployed

Figure 6 illustrates the experimental results for demand profile I when no recovery strategy is deployed.

Figure 6 depicts that demand placed at the factory drops by 50% during the pandemic and increases by 150% after the pandemic as compared to “old normal” demand which is considered 100%. The average service level at the end of simulation reaches 0.811 in the case in which no recovery measures are taken. In the inventory dynamics, we can observe a severe disruption resulting in almost zero inventory level in the supply chain, with some stabilization in the “new normal” during the pandemic.

**Fig. 7:**
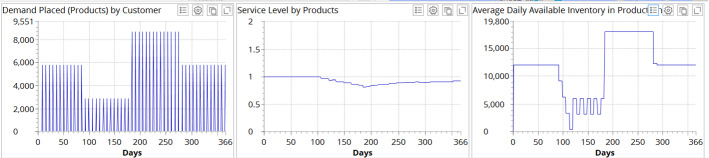
Experimental results for demand profile I and recovery strategy B (capacity ramp-up)

We then simulate demand profile I with the capacity ramp-up recovery strategy (Fig. 7).

We can observe a positive impact of the capacity ramp-up recovery strategy. Average service level is recovered by 0.918 by the end of the simulation period. Inventory dynamics follows the demand dynamics and so ensuring avoidance of disruption tails.

**Fig. 8:**
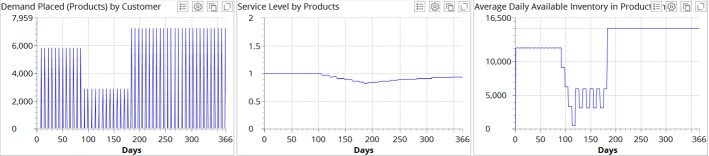
Experimental results for demand profile I and recovery strategies **a** (demand smoothing) and **b** (capacity ramp-up)

Subsequently, we simulate the impact of the combined deployment of both recovery strategies (i.e., demand smoothing and capacity ramp-up) (Fig. 8).

It becomes evident in Fig. 8 that supplementing the capacity ramp-up recovery strategy with the demand smoothing strategy has further positive effects that increase the service level to 0.931. Inventory dynamics follows the demand dynamics and adjusted capacity level which, as a synergetic effect allows for avoidance of disruption tails.

#### Profile II

**Fig. 9:**
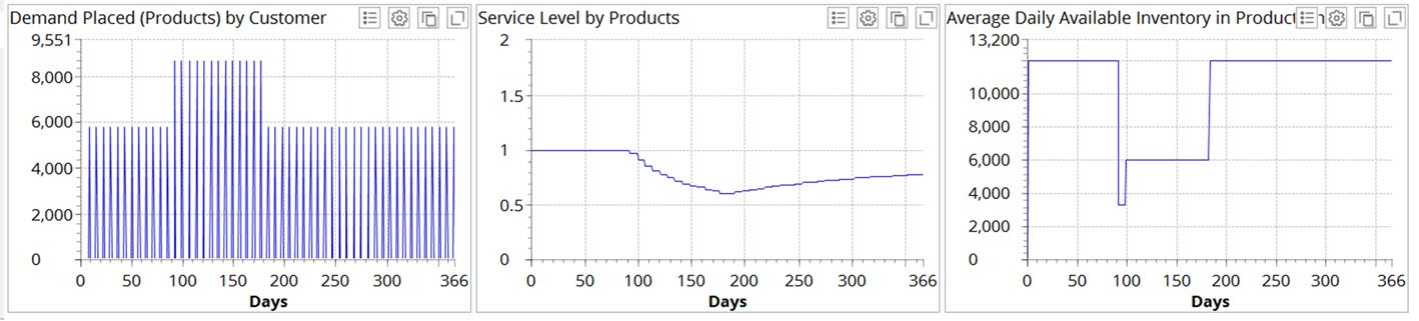
Experimental results for demand profile II when no recovery strategy is deployed

Figure 9 illustrates the experimental results for demand profile II when no recovery strategy is deployed.

Figure 9 depicts that demand placed at the factory increases by 50% during the pandemic and returns to an “old normal” level after the pandemic. The average service level at the end of simulation reaches 0.782 in the case in which no recovery measures are taken. In the inventory dynamics, we can observe a severe disruption in the supply chain during the pandemic.

**Fig. 10:**
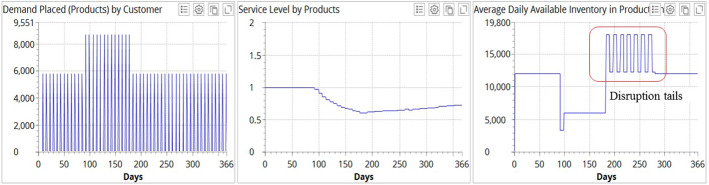
Experimental results for demand profile II and recovery strategy B (capacity ramp-up)

We then simulate demand profile II with the capacity ramp-up recovery strategy (Fig. 10).

Contrary to the simulation for demand profile I (cf. Figure 7), we observe a negative impact of the capacity ramp-up recovery strategy for demand profile II. Service level drops to 0.729. We elaborate on this and other simulation results, and their managerial implications in the next section.

## Theoretical and managerial insights

In this study, we analyzed supply chain management decisions for the time period of exiting a pandemic, with a particular focus on after-shock effects such as disruption tails. We used a discrete-event simulation model to investigate potential disruption tails at the time of exiting the COVID-19 pandemic. With the conceptualization and experimental part of our study, we contribute to both state-of-the-art and deduce some interesting managerial implications.

Theoretical insights from our study, for the first time, articulate an awareness of a novel and specific decision-making area in supply chain resilience related to exiting a pandemic and post-pandemic recovery. To date, we believe this is the first study that specifically considered that area. Our first major generalized theoretical insight is the existence of disruption tails as an after-shock risk associated with a pandemic. Second, we identified that demand dynamics, capacity management dynamics, and inventory-ordering control policies during the pandemic and in the course of exiting the pandemic are three major determinants which should be considered when modelling and managing supply chains at the stages of pandemic elimination and post-pandemic recovery.

Our study can inform managers about the existence and risk of disruption tails in their supply chains and be instructive for the selection of post-pandemic recovery strategies. In this section, we summarize the major findings obtained through our simulations and discuss their managerial implications (Table 3).

**Table 3:** Major findings and managerial implications

	Business-as-usual scenario	Disruption scenario I: demand decrease during and after a pandemic	Disruption scenario II: demand increase during a pandemic, and post-pandemic demand stabilization at the pre-pandemic level			
		Recovery strategies				
		No recovery strategy	Capacity increase during the period of exiting the pandemic	Capacity increase during the period of exiting the pandemic, and post-pandemic demand smoothing	No recovery strategy	Capacity increase during the period of exiting the pandemic
Service level	1.0	0.811	0.918	0.931	0.782	0.729
Managerial recommend-ations	Supply chain is efficient for business-as-usual scenarios	Supply chain coordination by means of demand smoothing in the post-pandemic period is an effective recovery measure that mediates the impact of disruption tails in the post-disruption period. In addition, gradual capacity ramp-up prior to expected peaks of postponed demand is an effective leveraging strategy for disruption tail control			It is not advisable to increase capacity in the case of demand profile II because of disruption tails. Recovery strategy B has a negative impact on the service level	

Our first observation is that if capacity and inventory management is unaware of the disruption tails, a highly destabilized production–inventory dynamic and performance decrease in the post-disruption period can be observed. Second, our results show that supply chains with “postponed demand” (i.e., backlog demand accumulated over the disruption period and shifted to the post-disruption period) and shutdown capacity during the COVID-19 pandemic (profile II) are particularly prone to disruption tails. We deployed our model to analyze strategies that can be used by firms in response to these disruption tails. With the simulation, we aimed to understand practical strategies to reduce the impact of disruption tails on supply chain networks. In particular, we developed and examined two strategies to avoid disruption tails. Supply chain coordination has been seen to be an effective recovery measure that mediates the impact of disruption tails by demand smoothing over time in the post-disruption period. In addition, gradual capacity ramp-ups prior to expected peaks of postponed demand have been found to be effective leveraging strategies for disruption tail control.

One practical way of implementing coordination is designing and using end-to-end supply chain visibility with the help of digital technologies such as artificial intelligence, blockchain, and supplier collaboration platforms (Brintrup et al. 2020; Dubey et al. 2021; Ivanov & Dolgui, 2020a; Ivanov et al. 2019, 2021; Nguyen et al. 2021; Sheng et al. 2021; Wamba & Queiroz, 2020; Winkelhaus & Grosse, 2020; Zouari et al. 2021). End-to-end visibility can ensure early disruption recognition, data transparency, and prediction of possible scenarios along with collaborative decision-making support.

Interestingly, in both profile I and profile II, we have observed disruption overlays. A disruption overlay is an effect of intersecting operational and disruption risks that have mutual impact on each other and amplify or dampen disruption propagations (Dolgui et al. 2020b). Ivanov (2020c) showed that “overlays occur if the negative consequences of changes in a supply chain as a result of a disruption are either amplified or mitigated by changes in the operational environment.” These overlays can be either *reciprocal* (i.e., complementary or mitigating) or *aggravate* (i.e., concurrent or enhancing). In profile I, we observed reciprocal overlays. Capacity disruption impact was mediated by simultaneous demand decline. In profile II, aggravate overlays have been observed because capacity disruption impact was enhanced by simultaneous demand increase. The analysis of the disruption overlays can therefore be useful for understanding the different reactions of supply chains in our simulation to different resilience strategies. For example, in demand profile II, it is not advisable to increase capacity because of disruption tails. Recovery strategy B even results in a negative impact on the service level because backlogged orders (i.e., orders accumulated but not fulfilled during the pandemic period) must be served in the post-pandemic recovery period, and new incoming orders must be delayed.

Finally, the results obtained can provide some recommendations for policy makers. Due to system inertia effects caused by different time lags of surges in demand and capacity adjustment, it might be instructive to inform supply chain companies about lockdown and re-opening times well in advance. That could help firms in managing shut-downing and ramping-up activities in the most efficient and responsive manner, and so avoiding disruption tails in particular and negative delayed effects of a pandemic in general. In addition, too frequent lockdown and re-opening decisions wreak havoc in supply chains due to their complexities, time lags and delayed effects in shutting-down and ramping-up the supply, production capacities, and logistics.

## Conclusion

Supply chains have experienced multiple shocks in the wake of the COVID-19 pandemic, both at its beginning and during the long-term period of deep uncertainty about short-term and long-term dynamics of demand and supply. While considerable efforts were invested in prediction of the pandemic’s impacts and examination of supply chain adaptive behaviors during the pandemic, little is known about supply chain management during pandemic elimination and post-disruption recovery periods. We took up this issue and developed a simulation study related to the exiting-the-pandemic period, which can be a challenging task because of after-shock effects such as disruption tails.

Our results (obtained for a specific and fragmented context) show that if capacity and inventory management is unaware of the disruption tails, this can result in highly destabilized production–inventory dynamics and performance decrease in the post-disruption period causing product deficits in the markets and high inventory costs in the supply chains. Our model can inform managers about the existence and risk of disruption tails in their supply chains. Our results show that supply chains with postponed demand and shutdown capacity during the COVID-19 pandemic are particularly prone to disruption tails. We developed and examined two strategies to avoid these disruption tails. First, we observed a conjunction of recovery and supply chain coordination, which mitigates the impact of disruption tails by demand smoothing over time in the post-disruption period. Second, we found that a gradual capacity ramp-up prior to expected peaks of postponed demand is an effective leveraging strategy for disruption tail control.

As with any study, limitations exist. Some of our study’s limitations are related to the modeling assumptions stated in Sect. 3. Our study also has a “classical” limitation of all simulation studies—that is, their contextual findings and limited generalizability. We assumed that supply chain structure does not change during the pandemic and in the post-pandemic periods. We did not consider demand variations during each of these periods to avoid unnecessary and inessential randomness, which would bias the output results and make them difficult to interpret correctly. We allowed for a capacity decrease and increase by some fixed level. The pandemic impact is modeled through surges in demand and capacity. Finally, the insights and findings described in this study are contextual and do not pretend to be generalizable. Our aim was to highlight the novel decision-making context of the post-pandemic recovery and illustrate some possible issues associated with this new decision-making setting.

The limitations stated above suggest directions for future research. For example, future research could explore settings in which some supply chain nodes completely disappear from the networks (e.g., because of supplier bankruptcy). Since we consider a case with one DC and one production facility, changes in supply chain structure cannot be applied to our experimental setting. Future research could also examine situations in which the capacity availability levels are dynamic and dependent on the pandemic dynamics (as captured by SIR modeling) and the forecasted number of infected employees. Specific pandemic control measures could be considered in more detail (e.g., the influence of the intensity of contacts and degree of mobility in society on the capacity and demand levels). Pre-pandemic decisions that could be made in anticipation of the pandemic could also be included in further simulations to examine the effects of firms’ early adaptive decisions over the course of the pandemic. Finally, other inventory and production control policies could be studied. Further efforts in these directions could enhance our understanding of effective management of super disruptions in supply chains due to pandemics.

## References

[CR1] Aldrighetti R, Battini D, Ivanov D, Zennaro I (2021). Costs of resilience and disruptions in supply chain network design models: a review and future research directions. International Journal of Production Economics.

[CR2] Aldrighetti R, Zennaro I, Finco S, Battini D (2020). Healthcare supply chain simulation with disruption considerations: A case study from Northern Italy. Global Journal of Flexible Systems Management.

[CR3] Alikhani R, Torabi SA, Altay N (2021). Retail supply chain network design with concurrent resilience capabilities. International Journal of Production Economics.

[CR4] Brintrup A, Pak J, Ratiney D, Pearce T, Wichmann P, Woodall P, McFarlane D (2020). Supply chain data analytics for predicting supplier disruptions: a case study in complex asset manufacturing. International Journal of Production Research.

[CR5] Carvalho H, Barroso AP, Machado VH, Azevedo A, Cruz-Mahado V (2012). Supply chain redesign for resilience using simulation. Computers & Industrial Engineering.

[CR6] Choi T-M (2020). Risk analysis in logistics systems: A research agenda during and after the COVID-19 pandemic. Transportation Research Part E: Logistics and Transportation.

[CR7] Choi T-M (2021). Fighting against COVID-19: What operations research can help and the sense-and-respond framework. Annals of Operations Research.

[CR8] Dolgui A, Ivanov D, Rozhkov M (2020). Does the ripple effect influence the bullwhip effect? an integrated analysis of structural and operational dynamics in the supply chain. International Journal of Production Research.

[CR9] Dolgui A, Ivanov D, Sokolov B (2018). Ripple effect in the supply chain: an analysis and recent literature. International Journal of Production Research.

[CR10] Dolgui A, Ivanov D, Sokolov B (2020). Reconfigurable supply chain: The X-network. International Journal of Production Research.

[CR11] Dubey R, Gunasekaran A, Childe SJ, Papadopoulos T, Blome C, Luo Z (2019). Antecedents of resilient supply chains: An empirical study. IEEE Transactions on Engineering Management.

[CR12] Dubey R, Gunasekaran A, Childe SJ, Wamba SF, Roubaud D, Foropon C (2021). Empirical Investigation of Data Analytics Capability and Organizational Flexibility as Complements to Supply Chain Resilience. International Journal of Production Research.

[CR13] El Baz J, Ruel S (2021). Can supply chain risk management practices mitigate the disruption impacts on supply chains’ resilience and robustness? Evidence from an empirical survey in a COVID-19 outbreak era. International Journal of Production Economics.

[CR14] Ghadge A, Dani S, Chester M, Kalawsky R (2013). A systems thinking approach for modelling supply chain risk propagation. Supply Chain Management: An International Journal.

[CR15] Golan MS, Jernegan LH, Linkov I (2020). Trends and applications of resilience analytics in supply chain modeling: Systematic literature review in the context of the COVID-19 pandemic. Environment Systems and Decisions.

[CR16] Govindan K, Mina H, Alavi B (2020). A decision support system for demand management in healthcare supply chains considering the epidemic outbreaks: A case study of coronavirus disease 2019 (COVID-19). Transportation Research Part E: Logistics and Transportation.

[CR17] Gupta V, Ivanov D, Choi T-M (2021). Competitive pricing of substitute products under supply disruption. Omega.

[CR18] He J, Alavifard F, Ivanov D, Jahani H (2019). A real-option approach to mitigate disruption risk in the supply chain. Omega The International Journal of Management Science.

[CR19] Hosseini S, Ivanov D, Blackhurst J (2020). Conceptualization and measurement of supply chain resilience in an open-system context. IEEE Transactions on Engineering Management.

[CR20] Hosseini S, Ivanov D, Dolgui A (2019). Review of quantitative methods for supply chain resilience analysis. Transportation Research: Part E.

[CR21] Ivanov D (2017). Simulation-based ripple effect modelling in the supply chain. International Journal of Production Research.

[CR22] Ivanov D (2018). Revealing interfaces of supply chain resilience and sustainability: a simulation study. International Journal of Production Research.

[CR23] Ivanov D (2021). Lean Resilience: AURA (Active Usage of Resilience Assets) Framework for Post-COVID-19 Supply Chain Management. International Journal of Logistics Management.

[CR24] Ivanov D (2021). Supply chain viability and the COVID-19 pandemic: A Conceptual and formal generalisation of four major adaptation strategies. International Journal of Production Research.

[CR25] Ivanov D (2021). Introduction to supply chain resilience.

[CR26] Ivanov D, Dolgui A (2021). OR-Methods for coping with the ripple effect in supply chains during COVID-19 pandemic: Managerial insights and research implications. International Journal of Production Economics.

[CR27] Ivanov D, Dolgui A (2020). A digital supply chain twin for managing the disruptions risks and resilience in the era of Industry 4.0. Production Planning and Control.

[CR28] Ivanov D (2019). Disruption tails and revival policies: A simulation analysis of supply chain design and production-ordering systems in the recovery and post-disruption periods. Computers and Industrial Engineering.

[CR29] Ivanov D (2020). Predicting the impact of epidemic outbreaks on the global supply chains: A simulation-based analysis on the example of coronavirus (COVID-19 / SARS-CoV-2) case. Transportation Research: Part E.

[CR30] Ivanov D (2020). Viable supply chain model: Integrating agility, resilience and sustainability perspectives. Lessons from and thinking beyond the COVID-19 pandemic. Annals of Operations Research.

[CR31] Ivanov D (2020). “A blessing in disguise” or “as if it wasn’t hard enough already”: Reciprocal and aggravate vulnerabilities in the supply chain. International Journal of Production Research.

[CR32] Ivanov D, Dolgui A (2020). Viability of intertwined supply networks: Extending the supply chain resilience angles towards survivability: A position paper motivated by COVID-19 outbreak. International Journal of Production Research.

[CR33] Ivanov D, Rozhkov M (2020). Coordination of production and ordering policies under capacity disruption and product write-off risk: An analytical study with real-data based simulations of a fast moving consumer goods company. Annals of Operations Research.

[CR34] Ivanov D, Dolgui A, Sokolov B (2019). The impact of digital technology and Industry 4.0 on the ripple effect and supply chain risk analytics. International Journal of Production Research.

[CR35] Ivanov D, Sokolov B, Chen W, Dolgui A, Werner F, Potryasaev S (2021). A control approach to scheduling flexibly configurable jobs with dynamic structural-logical constraints. IISE Transactions.

[CR36] Li Y, Chen K, Collignon S, Ivanov D (2021). Ripple effect in the supply chain network: Forward and backward disruption propagation, network health and firm vulnerability. European Journal of Operational Research.

[CR37] Li Y, Zobel CW, Seref O, Chatfield DC (2020). Network characteristics and supply chain resilience under conditions of risk propagation. International Journal of Production Economics.

[CR38] Llaguno A, Mula J, Campuzano-Bolarin F (2021). State of the art, conceptual framework and simulation analysis of the ripple effect on supply chains. International Journal of Production Research.

[CR39] Lohmer J, Bugert N, Lasch R (2020). Analysis of resilience strategies and ripple effect in blockchain-coordinated supply chains: An agent-based simulation study. International Journal of Production Economics.

[CR40] Lücker F, Chopra S, Seifert RW (2020) Mitigating product shortage due to disruptions in multi-stage supply chains. Production and Operations Management, forthcoming.

[CR41] Macdonald JR, Zobel CW, Melnyk SA, Griffis SE (2018). Supply chain risk and resilience: Theory building through structured experiments and simulation. International Journal of Production Research.

[CR42] Mehrotra S, Rahimian H, Barah M, Luo F, Schantz K (2020). A model of supply-chain decisions for resource sharing with an application to ventilator allocation to combat COVID-19. Naval Research Logistics.

[CR43] Nagurney A (2021). Supply chain game theory network modeling under labor constraints: Applications to the Covid-19 pandemic. European Journal of Operational Research.

[CR44] Nguyen S, Chen PS-L, Du Y (2021). Risk identification and modeling for blockchain-enabled container shipping. International Journal of Physical Distribution and Logistics Management.

[CR45] Paché G (2020). The “day after” covid-19 pandemic: logistical disorders in perspective. Review of European Studies.

[CR46] Paul SK, Chowdhury P (2021). A production recovery plan in manufacturing supply chains for a high-demand item during COVID-19. International Journal of Physical Distribution & Logistics Management.

[CR47] Queiroz MM, Ivanov D, Dolgui A, Fosso Wamba S (2020). Impacts of epidemic out-breaks on supply chains: Mapping a research agenda amid the COVID-19 pandemic through a structured literature review. Annals of Operations Research.

[CR48] Ruel S, El Baz J, Ivanov D, Das A (2021). Supply chain viability: Conceptualization, measurement, and nomological validation. Annals of Operations Research.

[CR49] Sawik T (2019). Two-period versus multi-period model for supply chain disruption management. International Journal of Production Research.

[CR50] Sawik T (2020). Supply chain disruption management.

[CR51] Schmitt TG, Kumar S, Stecke KE, Glover FW, Ehlen MA (2017). Mitigating disruptions in a multi-echelon supply chain using adaptive ordering. Omega.

[CR52] Schmitt AJ, Singh M (2012). A quantitative analysis of disruption risk in a multi-echelon supply chain. International Journal of Production Economics.

[CR53] Sheng J, Amankwah-Amoah J, Khan Z, Wang X (2021). COVID-19 pandemic in the new era of big data analytics: Methodological innovations and future research directions. British Journal of Management.

[CR54] Singh S, Kumar R, Panchal R, Tiwari MK (2021). Impact of COVID-19 on logistics systems and disruptions in food supply chain. International Journal of Production Research.

[CR55] Sodhi MM, Tang CS, Willenson E (2021). Research opportunities in preparing supply chains of essential goods for future pandemics. International Journal of Production Research.

[CR56] Tan WJ, Cai W, Zhang AN (2020). Structural-aware simulation analysis of supply chain resilience. International Journal of Production Research.

[CR57] Wamba, S.F., M.M. Queiroz (2020). Industry 4.0 and the supply chain digitalisation: a blockchain diffusion perspective. Production Planning & Control, 1–18

[CR58] Wieland A, Durach CF (2021). Two perspectives on supply chain resilience. Journal of Business Logistics.

[CR59] Wieland A (2021). Dancing the supply chain: Toward transformative supply chain management. Journal of Supply Chain Management.

[CR60] Winkelhaus S, Grosse EH (2020). Logistics 4.0: a systematic review towards a new logistics system. International Journal of Production Research.

[CR61] Xu S, Zhang X, Feng L, Yang W (2020). Disruption risks in supply chain management: A literature review based on bibliometric analysis. International Journal of Production Research.

[CR62] Yang J, Xie H, Yu G, Liu M (2021). Antecedents and consequences of supply chain risk management capabilities: An investigation in the post-coronavirus crisis. International Journal of Production Research.

[CR63] Zhao K, Zuo Z, Blackhurst JV (2019). Modelling supply chain adaptation for disruptions: An empirically grounded complex adaptive systems approach. Journal of Operations Management.

[CR64] Zouari D, Ruel S, Viale L (2021). Does digitalising the supply chain contribute to its resilience?. International Journal of Physical Distribution and Logistics Management.

